# Supplementary material for: Phenolic Compounds in Fractionated Blackcurrant Leaf Extracts in Relation to the Biological Activity of the Extracts
Source: Molecules. 2023 Nov 7;28(22):7459. doi: 10.3390/molecules28227459 (PMC10673464; doi:10.3390/molecules28227459)
Supplement: Supplementary file 1 [file molecules-28-07459-s001.zip › molecules-2682526-supplementary.pdf]

Table S-1. Calibration curves of phenolic compound standards used in quantitative HPLC-DAD analysis.

No	R_t [min]	Compound	standard curve equation	R^2
**9**	9.55	Gallic acid	y=56475x + 138773	0.999
**6**	10.20	Chlorogenic acid	y=61165x + 49914	0.9907
**7**	11.10	Neochlorogenic acid	y=22107x-5174	0.9997
**18**	16.45	*p*-Coumaric acid	y=16141x - 52192	0.9999
**13**	17.51	Quercetin-3-*O*-rutinoside	y=40995x - 592911	0.9975
**20**	17.99	Ferulic acid	y=30793x + 112696	0.9999
**22**	18.28	Kaempferol 7-*O*-glucoside	y=23945x-8717	0.9998
**15**	18.39	Quercetin-3-*O*-glucoside	y=49665x - 432618	0.9996
**16**	19.17	Quercetin-3-*O*-galactoside	y=48462x + 681014	0.9999
**19**	21.01	Quercetin 3-*O*-malonylglucoside	y=14488x+18.5	0.9995
**11**	22.53	Rosmarinic acid	y= 26003x + 859322	0.9940
**12**	33.19	Quercetin	y=44008x - 84195	0.9906

Table S-2. Spearman's correlation coefficients analysis.

Y	X	r	r	t	Pr(>|t|)
ABTS	11	0,982	0,9643	16,4408	0
ABTS	20	0,978	0,9566	14,8417	0
ABTS	13	0,9698	0,9406	12,5818	0
ABTS	22	0,9293	0,8637	7,9593	0
DPPH	11	0,9291	0,8632	7,944	0
PC-3	9	0,9242	0,8541	7,6524	0
DPPH	13	0,9234	0,8527	7,6081	0
ABTS	15	0,9142	0,8357	7,1319	0
DPPH	15	0,9095	0,8272	6,9179	0
DPPH	22	0,9024	0,8143	6,6217	0,0001
DPPH	16	0,8975	0,8056	6,4373	0,0001
ABTS	16	0,8904	0,7928	6,1862	0,0001
DPPH	20	0,8899	0,7918	6,1676	0,0001
PC-3	6	0,7354	0,5409	3,4323	0,0064
ABTS	7	0,708	0,5013	3,1707	0,01
DPPH	18	0,6537	0,4273	2,7316	0,0211
DPPH	7	0,6314	0,3987	2,5748	0,0277
ABTS	18	0,6145	0,3776	2,4632	0,0335
HTC116	12	0,5263	0,2769	1,9571	0,0788
HTC116	18	-0,5249	0,2755	-1,95	0,0797
HTC116	7	-0,7254	0,5262	-3,3323	0,0076
HTC116	22	-0,7518	0,5653	-3,606	0,0048
HTC116	16	-0,8007	0,6412	-4,2272	0,0018
HTC116	15	-0,8157	0,6653	-4,4588	0,0012

HTC116	11	-0,8901	0,7922	-6,1748	0,0001
HTC116	13	-0,9067	0,8222	-6,7999	0
HTC116	20	-0,9294	0,8637	-7,9601	0
